# Two New Species of the Genus *Philanthaxia* Deyrolle, 1864, from Hainan Province, China (Coleoptera, Buprestidae, Thomassetiini) †

**DOI:** 10.3390/insects16080839

**Published:** 2025-08-14

**Authors:** Tao Ni, Zhihao Qi, Xiancheng Peng, Haitian Song, Rong Wang

**Affiliations:** 1 State Key Laboratory of Agricultural and Forestry Biosecurity, College of Forestry, Fujian Agriculture and Forestry University, Fuzhou 350002, China; 12204290l0@fafu.edu.cn (T.N.); qizhihao2021@126.com (Z.Q.); 13358295087@163.com (X.P.); 2 Fujian Academy of Forestry, Fuzhou 350012, China

**Keywords:** taxonomy, buprestinae, *Philanthaxia*, new species, Hainan

## Abstract

In this paper, we describe two new species of *Philanthaxia* Deyrolle, 1864, from Hainan, China, namely, *P. longicornis* Ni & Song, sp. nov., and *P. lui* Ni & Song, sp. nov., with the adult host plant *Casearia membranacea* Hance (Salicaceae). The characters of these new species are described with illustrations, comparisons are made with the most similar species, and the COI gene sequence of *P. longicornis* and its analysis are provided.

## Simple Summary

Two new species of the genus *Philanthaxia* were identified in Hainan, China. Morphological comparisons with existing species demonstrated significant taxonomic distinctions, confirming their status as new taxa. The adult host plant of both novelties is *Casearia membranacea* Hance (Salicaceae).

## 1. Introduction

The genus *Philanthaxia* Deyrolle, 1864, belongs to the tribe Thomassetiini Bellamy, 1987, and was described with *Philanthaxia curta* Deyrolle, 1864, as the type species [1]. Species of the genus *Philanthaxia* are characterized by smaller, more oval-shaped eyes and a more convex vertex, which is typically 4.5–6.0 times wider than the eye width; the pronotum being widest at the base, with dense reticulate or punctate sculpturing; and the anal ventrite being regularly rounded, roundly truncated or rarely incurved apically [2].

Before the first revision by Bílý in 1993, *Philanthaxia* was mainly considered to be distributed in South and Southeast Asia as well as the Greater Sunda Islands [2]. Subsequently, the distribution of this genus expanded, with new records from the Maluku Islands and Irian Jaya (Papua) [3,4]. In China, only two species of the genus have been recorded in the Taiwan region: *Philanthaxia sauteri* Kerremans, 1912 [5], and *Philanthaxia convexifrons* Kurosawa, 1954 [6]. In this paper we provide a comparison between these two known species and two species new to science.

At present, records of the biology of the genus *Philanthaxia* are poorly known. The existing records only report that the adults of *Philanthaxia* species are found on *Castanopsis* sp., primarily in the higher level of the canopy, and larvae of this genus are still unknown [7].

Following Bílý’s revision in 1993, Bellamy’s 2008 catalogue documented 61 valid species within *Philanthaxia* [8]. Taking into account 4 new species described by Bílý & Nakládal [7], 4 new species described by Bílý [9], 1 new species reported by Ohmomo [10], and 2 new species described herein from Hainan, China, the total number of species in this genus has increased to 72 worldwide, including 1 fossil species [11].

## 2. Materials and Methods

### 2.1. Insect Collection and Image Processing

**Figure 1:**
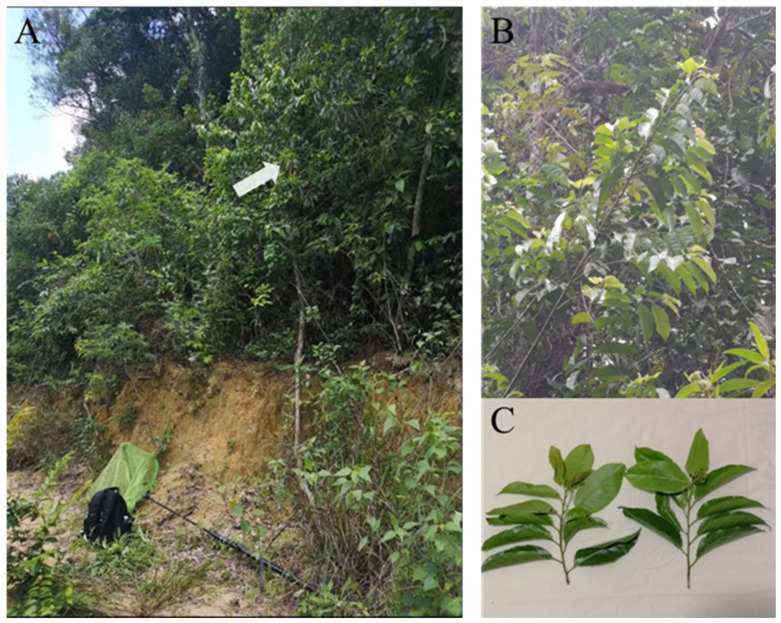
(**A**) Collecting site photographed at Lizhiling. The arrow indicates the host plant *Casearia membranacea*. (**B**,**C**) Host plant leaves.

**Figure 2:**
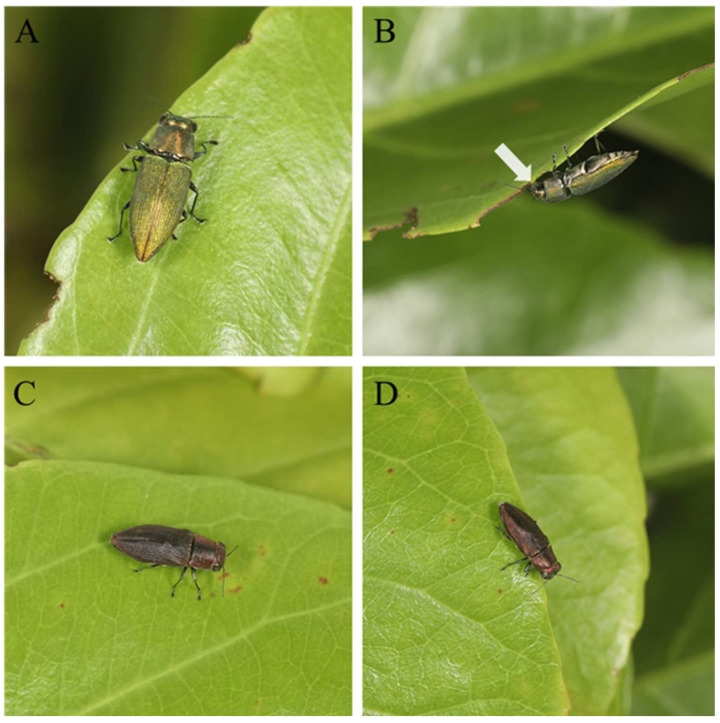
(**A**,**B**) *Philanthaxia longicornis* Ni & Song, sp. nov., feeding on leaves; the arrow indicates the specific feeding site. (**C**,**D**) *Philanthaxia lui* Ni & Song, sp. nov., staying on leaves.

The two new species described in this article were captured on the leaves of *Casearia membranacea* Hance (Salicaceae) in Lizhiling, Sanya, Hainan (Figure 1 and Figure 2). Using standardized aerial sweep nets (Bingqing trading, Model: HL-PW), we implemented systematic sweep netting across all accessible strata of tall-canopied *Casearia* trees (height > 6 m). Each tree received 20 pendulum sweeps at 3 vertical levels (understory: 0–2 m; mid-canopy: 2–4 m; upper-canopy: 6 m+ via telescopic pole).

The photos were obtained using a Canon EOS 5DmarkIV (Canon, Beijing, China) with an EF70-200 mm f2.8 IS II USM lens (Canon, Beijing, China) for Figure 1 and an EF 100 mm f2.8L IS USM lens (Canon, Beijing, China) for Figure 2.

The specimens were studied using a Keyence VHX-5000 digital microscope (Keyence, Tokyo, Japan) with a VH-Z20R zoom lens (Keyence, Tokyo, Japan), images were processed and the layers combined into figures using Adobe Photoshop CC 2018. The measurements were followed by Bílý [9] and Qi [12] and made as follows:

Body length: Length between the top of the head and the tip of the elytra.

Body width: Widest part of the body.

Aedeagus length: Length between the base and the tip of the aedeagus.

Aedeagus width: The widest part of the parameres.

### 2.2. DNA Extraction and Sequence Analysis

Use TIANamp Micro DNA Kit (DP316, Tiangen, Beijing, China) to extract DNA, and refer to the instructions for specific experimental steps. Then use primers [13] (LCO1490: 5′-GGTCAACAAATCATAAAGATATTGG-3′, HCO2198: 5′-TAAACTTCAGGGTGACCAAAAAATCA-3′) for PCR amplification and sequence the PCR products.

Use DAMBE (ver. 7.3.32) to calculate ISS and ISS.C values for the target sequence with all 22 species’ sequences of the Bupretidae family on NCBI, and use Mega (ver. 7.0) to align construct evolutionary tree (Maximum Likelihood, ML) based on the Kimura 2-parameter genetic distance model.

All specimens examined herein are housed in the collection of Fujian Academy of Forestry, Fuzhou, China (FAF).

## 3. Results

Taxonomy

Family Buprestidae Leach, 1815;

Subfamily Buprestinae Leach, 1815;

Tribe Thomassetiini Bellamy, 1987;

Genus *Philanthaxia* Deyrolle, 1864;

Type species: *Philanthaxia curta* Deyrolle, 1864.

### 3.1. Philanthaxia longicornis Ni & Song, sp. nov.

http://zoobank.org/urn:lsid:zoobank.org:act:ECC1A5B8-75A0-4C1E-B081-F3D0935D940C

(Figure 3)

Type locality: Mt. Lizhiling, Sanya City, Hainan Province, China.

Type specimen: Holotype (male, FAF): Southern Hainan Province, Sanya City, Mt. Lizhiling, 18°21′21″ N 109°27′20″ E, alt. 390 m, 2021-VI-19, Haitian Song leg.; paratypes (15 males, 9 females, FAF; 1 male (NACRC: IOZ (E)224851), 1 female (NACRC: IOZ (E) 224852): 18°21′21″ N 109°27′20″ E, alt. 390 m, 2021-VI-19, Haitian Song leg.; 2021-VI-22, Lu leg.; 2022-V-23, Jiasheng Lu leg.; 2023-VI-1, Shijie Lu leg.

Diagnosis: Medium-sized, rather convex, dorsal and ventral surfaces golden-green with metallic luster, sides of pronotum slightly depressed, frons with fine white setae and with extremely long antennomeres.

Description of holotype: Length 6.9 mm, width 2.6 mm; the aedeagus measures around 2.4 mm in length and 0.4 mm in width. Spindle-shaped, entirely golden-green with metallic luster.

Head (Figure 5A) golden-green, broader than the lateral side of pronotum, covered with evenly small, reticulate, irregular polygonal sculpturing. Fine white setae present at the frons. The vertex relatively flat, 5.1 times wider than the eye. The eyes large, nearly oval and laterally convex. The antennae (Figure 5E) very slender and long, reaching beyond elytral base and scutellum, consisting of 11 segments, with white setae. The scape rod-shaped, 6.0 times longer than wide. The pedicel short and ovoid, 2.0 times as long as wide. Antennomere 3 almost cylindrical, 2.4 times as long as wide. Antennomeres 4–10 slender and triangular, with a length-to-width ratio of 2.1–3.5; antennomeres 4 and 5 longer and the distal 2.0 times shorter. The terminal antennomere nearly triangular, 1.5 times longer than wide.

Pronotum (Figure 5A) nearly trapezoidal, about 1.6 times wider than long, covered with the same sculpturing as that on the head. Overall golden-green with a metallic sheen. The anterior edge curved, slightly convex in the middle; the posterior edge nearly straight, with the sides distinctly converging forward, slightly curved. The scutellum wide, 2.0 times as wide as long, dark green, subcordiform and depressed near the base of the pronotum.

Elytra (Figure 5C) 1.8 times longer than wide, with yellow coloration on both sides of the elytral suture, tapers sharply near the distal third. Each elytron has eight distinct striae with intervals bearing fine transverse rugosities. The lateral margins with fine serrations along apical third. The humeral callosities do not prominently protrude beyond the elytral lateral edges.

**Figure 3:**
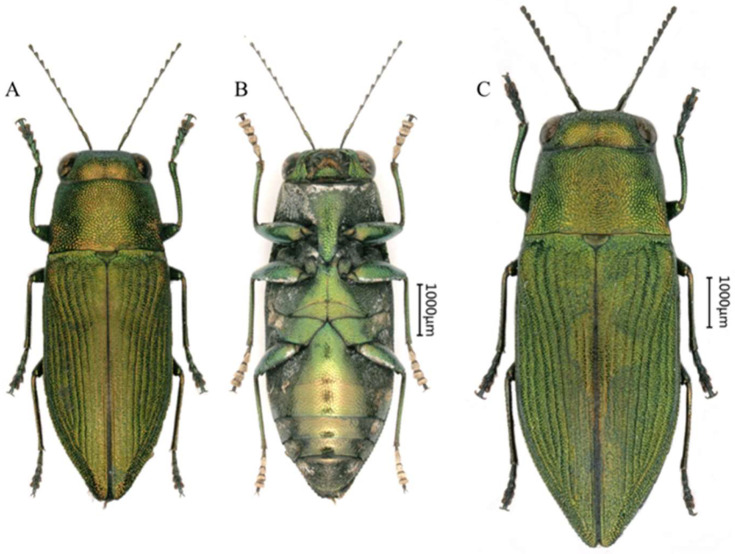
Habitus of *Philanthaxia longicornis* Ni & Song, sp. nov. (**A**,**B**) Male, holotype, 6.9 mm, from Sanya, Hainan. (**C**) Female, paratype, 8.4 mm, from Sanya, Hainan. (**A**,**C**) Dorsal view. (**B**) Ventral view. Scale bar = 1.0 mm.

Legs (Figure 3) slender, covered with setae; fore tibiae slightly bent outward, without serrations, and tarsal segments 2–4 enlarged. The tarsal segments covered with setae.

**Figure 5:**
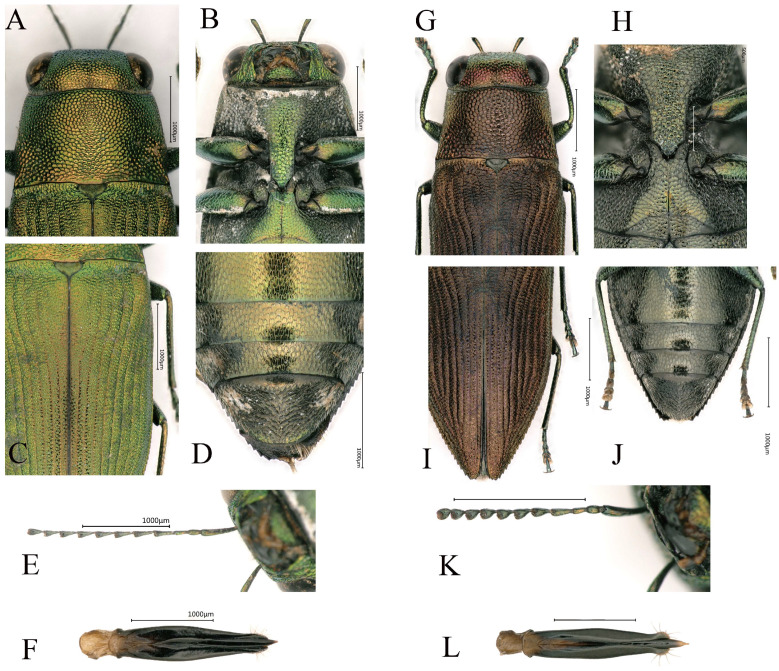
(**A**–**F**) *Philanthaxia longicornis* Ni & Song, sp. nov. (holotype). (**G**–**L**) *Philanthaxia lui* Ni & Song, sp. nov. (holotype). (**A**,**G**) Details of pronotum. (**B**,**H**) Prosternal process. (**C**,**I**) Elytra. (**D**,**J**) Abdomen. (**E**,**K**) Antennae. (**F**,**L**) Aedeagus. (**A**,**C**,**F**,**G**,**I**,**L**) Dorsal view. (**B**,**D**,**E**,**H**,**J**,**K**) Ventral view. Scale bar = 1.0 mm, except scale bar of (**H**) = 0.5 mm.

Ventral side (Figure 3B) of the abdomen sparsely covered with short white setae. Prosternal process (Figure 5B) elongated, angularly tapering at apex. Abdominal apex lighter, changing from green to gold, with darker sides changing from green to black. The ventral surface of the last visible ventrite (Figure 5D) more or less black, covered with relatively denser and longer white setae, apex rounded.

Aedeagus (Figure 5F) widest at the middle, tapering at the posterior half; apices of parameres sharp, not distinctly expanded, the sides of the terminal portion covered with setae; apex of median lobe sharp.

Sexual dimorphism: Female (Figure 3C) clearly differs from male by the much shorter antennae and somewhat more robust body.

Etymology: This species is named after its morphological character of long antennae.

Differential diagnosis: The differential diagnosis is provided in Table 1.

Distribution: China (Hainan).

### 3.2. Philanthaxia lui Ni & Song, sp. nov.

http://zoobank.org/urn:lsid:zoobank.org:act:6D1955AD-DF22-46C6-8D63-33F1403945E9

(Figure 4)

Type locality: Mt. Lizhiling, Sanya City, Hainan Province, China.

Type specimen: Holotype (male, FAF): Southern Hainan Province, Sanya City, Mt. Lizhiling, 18°21′21″ N 109°27′20″ E, alt. 390 m, 2021-VI-19, Haitian Song leg.; paratypes (1 female, FAF; 1 female (NACRC: IOZ (E) 224853.) same data as holotype.

Diagnosis: Medium-sized, dorsally green, ventrally bronze, with metallic luster, pronotum evenly convex, frons green with fine setae.

Description of holotype: Length 5.6 mm, width 2.1 mm; the aedeagus measures around 2.2 mm in length and 0.3 mm in width. Fusiform with most of the anterior part of the body copper-colored with metallic luster.

Head (Figure 5G) large and broad, significantly wider than the pronotum. Vertex flat without noticeable convexity, and width about 4.8 times the eye width. Eyes large, oval and prominently protruding beyond the head sides. The sculpture consists of small, irregular polygonal cells with clearly defined boundaries and a metallic copper-green sheen. A fine groove runs along the middle, and the apex is covered with extremely fine white setae. Antennae (Figure 5K) long, almost reaching the base of the pronotum; scape nearly clavate, slightly curved and 5.7 times longer than wide; pedicel ovoid, about twice as long as wide. Antennomere 3 slender and ovoid, about 5.0 times as long as wide. Antennomeres 4–10 transitional from conical to nearly triangular in shape, shorter and thicker, with lengths 2.0–2.4 times their widths. The terminal flagellomere nearly triangular, 2.0 times as long as wide.

Pronotum (Figure 5G) evenly convex, 1.4–1.7 times as wide as long; anterior margin convex at the center, posterior margin slightly curves inward at middle, lateral margins mostly straight. The sides of the pronotum bear paired symmetrical shallow depressions. The posterolateral edges slightly expand outward, marking the widest point of the pronotum. The pronotum sculpture resembles that of the head but with larger individual sculptural cells. Scutellum large, darker in color than the pronotum, subcordiform, depressed at the anterior, 1.6 times as wide as long.

Elytra (Figure 5I) flat, slightly raised along the suture, 2.0 times as long as wide; two-thirds of the anterior part nearly parallel, with fine serrations along the sides, while the posterior third tapers sharply, with serrations larger than those on the anterior two-thirds. The humeral callosities distinct, with the apex slightly projecting beyond the sides of the elytra. The basal transverse area broadly and shallowly depressed, almost connecting with the scutellum, and aligns with the pronotum without notable indentations or protrusions. Each elytron has eight deep and distinct longitudinal striae, with intervals bearing fine transverse rugosities.

Ventral side (Figure 5E) overall dark green, with the abdominal ventrites being the lightest in color. The entire abdomen flat and covered with fine setae. Prosternal process (Figure 5H) broad, nearly parallel and has an obtusely pointed apex. The anal ventrite (Figure 5J) nearly circular apically, lacks serrations and covered with setae.

Legs (Figure 5D,E) slender, covered with setae, without serration, fore tibiae with gentle bent outward and the anterior tarsal claws hooked.

Aedeagus (Figure 5L) slender, subparallel. Parameres widest at the middle, converging from basal 2/3 of length, apical everted and bearing setae. Apex of median lobe transparent and needle-shaped.

Sexual dimorphism: Female differs from male by its much larger size and reddish frons and lateral sides of pronotum.

Etymology: This new species is named after Mr. Jiasheng Lu (Sanya, Hainan), who is a Lepidoptera enthusiasts, leading the way for us during the collection process.

Differential diagnosis: The differential diagnosis against two congeners is provided in Table 2.

Distribution: China (Hainan).

### 3.3. Molecular Identification of Philanthaxia longicornis Ni & Song, sp. nov.

#### 3.3.1. DAMBE Base Sequence Analysis

Sequence evolutionary analysis was performed using DAMBE v7.3.32 on the 23 obtained sequences. The calculated index of ISS = 0.3632 was significantly lower than the ISS.C = 0.7850 (*p* < 0.001). This confirms minimal substitution saturation, validating the suitability of these sequences for robust phylogenetic reconstruction.

#### 3.3.2. Genetic Distance Analysis

The genetic distances among the 23 sequences were analyzed using the Kimura two-parameter model with bootstrap testing (1000 replicates). As shown in Table 3, interspecific genetic distances ranged from 0.137 to 0.308, with a mean value of 0.2504. The smallest genetic distance (0.196) was observed between *P. longicornis* Ni & Song, sp. nov. and *Chrysochroa fulgidissima*, whereas the largest distance (0.308) was recorded between *P. longicornis* Ni & Song, sp. nov. and *Endelus continentalis,* indicating significant differences in genetic distance among different species. This sequence can be used as a basis for analyzing and identifying species.

#### 3.3.3. Phylogenetic Analysis

**Figure 6:**
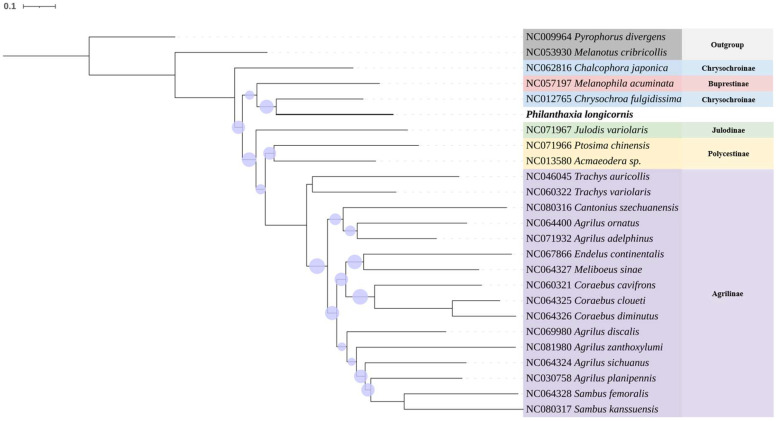
Maximum Likelihood tree based on the analysis of COⅠ gene sequences of *Philanthaxia longicornis* Ni & Song, sp. nov. and Buprestidae species. The purple dots represent the bootstrap value (15–95),the larger the dots, the higher the value. Outgroup: *Pyrophorus divergens* (Elateridae) and *Melanotus cribcollis* (Elateridae). Rooted at *P. divergens* clade.

The phylogenetic tree was constructed using the Maximum Likelihood method with the Kimura two-parameter genetic distance model, and branch confidence was assessed via 1000 bootstrap replicates. As illustrated in Figure 6, *P. longicornis* Ni & Song, sp. nov. exhibits significantly lower bootstrap values compared to other species, indicating greater genetic divergence. In contrast, conspecific specimens within the same genus display bootstrap values approaching unity, consistent with closer genetic relationships.

## 4. Discussion

*Philanthaxia longicornis* Ni & Song, sp. nov. and *P*. *lui* Ni & Song, sp. nov. represent the first record of this genus on the Chinese mainland. We have further extracted the COI gene sequence from *P. longicornis* and conducted evolutionary analyses through comparative sequencing with COI genes from 22 other species within the family Buprestidae and 2 outgroup species. The genetic distance and bootstrap value indicate that the molecular identification results corroborated the morphological identification findings, and added a COI sequence to the genus *Philanthaxia*, which is the first record of molecular data in this genus. The results of the COI phylogenetic analysis show that *P. longicornis* is closer to the species of subfamily Buprestidae and Chrysochroinae compared to Julodinae and Polycestinae, while compare to subfamily Buprestidae, *P. longicornis* is closer to the species of subfamily Chrysochroinae, and *Melanophila acuminata* is predicted between two species of Chrysochroinae, we speculate that subfamily Buprestidae and Chrysochroinae have a highly homologous, it is similar to previous research results [14]. The other subfamilies showed clear clustering relationships within the same subfamily and distinct species segregation. Most of the analysis based on the COⅠ gene sequence is consistent with the results of genome analysis in previous studies, such as Julodinae being closest to Polycestinae with high bootstrap values, which is consistent with the results of a previous study [14], and Agrilinae has a significant genetic distance from other subfamilies without species crossing, which is consistent with previous studies [14,15,16]. There are also differences from previous studies, such as the fact that the two species of the genus *Trachys* did not cluster in the closer clade; these differences only appear in the subfamily Agrilinae [17].

Owing to the limited number of *P. lui* paratype specimens collected, molecular analysis was not performed on this species in the present study. Based on the concordant morphological and molecular identification results obtained for *P. longicornis*, we consider the morphological identification of *P. lui* to be reliable. Future collection of additional specimens will facilitate the acquisition of complementary molecular data.

Additionally, given comparable climatic conditions and historically limited surveys, we hypothesize potential occurrences of *Philanthaxia* in other southern Chinese provinces (e.g., Yunnan, Fujian, Guangdong). Verification requires systematic long-term field investigations across these regions.

## Figures and Tables

**Figure 4:**
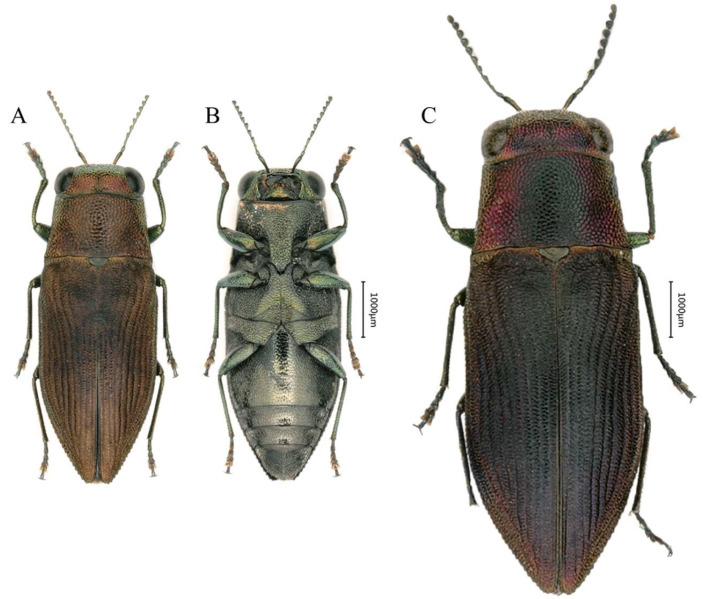
Habitus of *Philanthaxia lui* Ni & Song, sp. nov. (**A**,**B**) Male, holotype, 5.6 mm, from Sanya, Hainan. (**C**) Female, paratype, 8.5 mm, from Sanya, Hainan. (**A**,**C**) Dorsal view. (**B**) Ventral view. Scale bar = 1.0 mm.

**Table 1:** Differential diagnosis of *Philanthaxia longicorna* Ni & Song, sp. nov., with two morphologically similar congeners.

	*Philanthaxia splendida*, Van De Poll, 1892 (Vietnam)	*Philanthaxia sauteri* Kerremans, 1912 (Taiwan, China)	*Philanthaxia longicornis* Ni & Song, sp. nov. (Hainan, China)
Size	5.0–7.2 mm	8.0–10.0 mm	6.7–7.9 mm
Color	Bright golden-green; no record of ventral side color	Dark color, mainly green, with red or black; ventral side green or reddish	Bright golden-green, same on ventral side
Head	Frons less convex. Eyes rather large and strongly projecting beyond outline of head.	Frons slightly depressed. Eyes rather small and slightly projecting beyond outline of head.	Frons evenly convex. Eyes middle size and projecting beyond outline of head.
Antenna	Antennae reach two-thirds of pronotum; length–width ratio: scape 3.3, pedicel 1.7, antennomeres 3–10 1.4–2.8, terminal 1.6	Antennae reach half of pronotum; length–width ratio: scape 2.8, pedicel 1.9, antennomeres 3–10 1.2–1.5, terminal 1.3	Antennae extremely long, reaching beyond elytral base and scutellum; length–width ratio: scape 6.0, pedicel 2.0, antennomeres 3–10 2.1–3.5, terminal 1.5
Pronotum	Generally evenly convex on both sides; sculpture regular, dense near the base	Depressed laterally near posterior angles; sculpture forming irregular horizontal pattern	Both sides slightly depressed at base; sculpture regular polygon, dense near the base
Elytra	Golden-green, monochromatic	Golden-green along suture but color fading or changing reddish or violet toward sides	Golden-green, monochromatic
Aedeagus	Relatively short; parameres separated at one-sixth of their length from base with apical portion raised	Relatively robust; parameres separated in posterior half without being raised	Relatively slender; parameres separated at about one-quarter, with apical portion raised

**Table 2:** Differential diagnosis of *Philanthaxia lui* Ni & Song, sp. nov., with two morphologically similar congeners.

	*Philanthaxia* *convexifrons* Kurosawa, 1954 (Taiwan, China)	*Philanthaxia iriei* Kurosawa, 1985 (Ryukyu)	*Philanthaxia lui* Ni & Song sp. nov. (Hainan, China)		*Philanthaxia* *splendida*, Van De Poll, 1892 (Vietnam)	*Philanthaxia* *sauteri* Kerremans, 1912 (Taiwan)	*Philanthaxia* *longicorna* Ni & Song, sp. nov. (Hainan)
Size	6.9–7.0 mm	6.8–8.0 mm	5.6–8.6 mm	Size	5.0–7.2 mm	8.0–10.0 mm	6.7–7.9 mm
Color	Bronze-black; ventral side bronze-black	Bronze-black or reddish-bronze; no record of ventral side color	Bronze-black; ventral side dark green	Color	Bright golden-green; no record of ventral side color	Dark color, mainly green, with red or black; ventral side green or reddish	Bright golden-green, same with ventral side
Head	Frons evenly convex without any depressions or reliefs, same color in both sexes; smaller eyes, slightly projecting beyond outline of head	Frons slightly convex between eyes, female frons bronze or reddish, male frons green; larger eyes, strongly projecting beyond outline of head	Frons evenly convex, female frons reddish, male frons green; eyes large, projecting beyond outline of head	Head	Frons less convex, larger head and eyes	Frons slightly depressed, smaller head and eyes	Frons evenly convex, larger head and eyes
Pronotum	Generally evenly convex, bearing regular and small polygonal sculpture	Generally evenly convex, sculpture horizontally irregular, dense near the base	Both sides of base slightly depressed, with more regular and large polygonal sculpture	Antenna	Antennae reach two third of pronotum horizontally; length-width ratio: scape 3.3, pedicel 1.7, antennae 3–10 1.4–2.8, terminal 1.6	Antennae reach half of pronotum horizontally; length/width ratio: scape 2.8, pedicel 1.9, antennae 3–10 1.2–1.5, terminal 1.3	Antennae extremely long, reach elytra and over scutellum horizontally; length/width ratio: scape 6, pedicel 2.0, antennae 3–10 2.1–3.5, terminal 1.5
Elytra	Black, slightly violet, monochromatic	Bronze-black and reddish, shiny, with serrations along lateral sides	Bronze-black, monochromatic, with serrations at posterior third of lateral sides	Pronotum	Generally evenly convex on both sides; sculpture regular, dense near the base	Depressed laterally near posterior angle; sculpture irregular horizontal pattern	Both sides of basal slightly depressed; sculpture regular polygon, dense near the base

**Table 3:** Inter-species genetic distance of Buprestidae family insects and *P. longicornis* Ni & Song, sp. nov. based on Kimura 2-parameter model.

	1	2	3	4	5	6	7	8	9	10	11	12	13	14	15	16	17	18	19	20	21	22	23
1		0.020	0.020	0.020	0.023	0.022	0.021	0.025	0.024	0.021	0.019	0.022	0.020	0.023	0.020	0.024	0.021	0.021	0.025	0.020	0.022	0.020	0.021
2	0.196		0.013	0.015	0.015	0.014	0.014	0.016	0.015	0.015	0.013	0.015	0.012	0.014	0.012	0.015	0.012	0.014	0.015	0.014	0.013	0.013	0.014
3	0.236	0.241		0.015	0.015	0.016	0.016	0.018	0.017	0.016	0.015	0.016	0.015	0.015	0.015	0.015	0.015	0.016	0.017	0.016	0.017	0.015	0.015
4	0.222	0.246	0.258		0.013	0.013	0.014	0.014	0.015	0.014	0.012	0.013	0.014	0.013	0.013	0.015	0.015	0.014	0.014	0.014	0.014	0.015	0.015
5	0.267	0.258	0.275	0.243		0.015	0.016	0.013	0.014	0.014	0.015	0.012	0.015	0.016	0.015	0.015	0.015	0.014	0.016	0.014	0.015	0.016	0.013
6	0.272	0.248	0.281	0.210	0.263		0.014	0.016	0.016	0.014	0.014	0.014	0.015	0.013	0.014	0.017	0.015	0.013	0.015	0.016	0.015	0.016	0.015
7	0.259	0.252	0.271	0.242	0.270	0.241		0.015	0.015	0.014	0.013	0.015	0.015	0.014	0.014	0.016	0.013	0.014	0.015	0.013	0.015	0.016	0.015
8	0.289	0.287	0.280	0.260	0.216	0.274	0.258		0.010	0.015	0.014	0.014	0.015	0.016	0.016	0.015	0.015	0.014	0.015	0.017	0.015	0.016	0.015
9	0.271	0.259	0.279	0.251	0.224	0.266	0.271	0.137		0.015	0.014	0.016	0.016	0.015	0.017	0.016	0.015	0.016	0.016	0.016	0.016	0.016	0.017
10	0.246	0.265	0.266	0.224	0.223	0.238	0.239	0.249	0.251		0.014	0.014	0.014	0.014	0.016	0.016	0.013	0.012	0.016	0.013	0.014	0.015	0.014
11	0.215	0.208	0.244	0.206	0.257	0.245	0.241	0.268	0.263	0.231		0.013	0.015	0.014	0.014	0.014	0.014	0.014	0.015	0.014	0.014	0.015	0.015
12	0.268	0.256	0.274	0.230	0.225	0.239	0.251	0.271	0.267	0.216	0.230		0.014	0.013	0.015	0.016	0.014	0.013	0.016	0.014	0.013	0.016	0.012
13	0.243	0.237	0.246	0.220	0.254	0.254	0.255	0.265	0.261	0.212	0.242	0.239		0.013	0.014	0.016	0.013	0.012	0.014	0.013	0.012	0.014	0.013
14	0.267	0.266	0.302	0.231	0.282	0.243	0.254	0.305	0.278	0.240	0.248	0.252	0.252		0.015	0.016	0.013	0.013	0.015	0.014	0.014	0.016	0.014
15	0.235	0.224	0.264	0.234	0.283	0.261	0.244	0.288	0.289	0.261	0.230	0.265	0.247	0.278		0.014	0.014	0.014	0.015	0.015	0.014	0.015	0.014
16	0.260	0.253	0.253	0.273	0.293	0.296	0.264	0.261	0.280	0.280	0.241	0.303	0.286	0.296	0.272		0.014	0.016	0.018	0.014	0.017	0.015	0.016
17	0.247	0.230	0.261	0.231	0.235	0.233	0.224	0.254	0.250	0.206	0.234	0.226	0.221	0.234	0.255	0.255		0.013	0.014	0.013	0.013	0.014	0.013
18	0.232	0.243	0.271	0.226	0.246	0.224	0.234	0.250	0.253	0.188	0.240	0.206	0.224	0.243	0.251	0.274	0.212		0.014	0.014	0.012	0.016	0.013
19	0.308	0.279	0.291	0.252	0.287	0.279	0.252	0.277	0.295	0.267	0.262	0.282	0.250	0.272	0.270	0.290	0.248	0.236		0.015	0.015	0.017	0.016
20	0.256	0.260	0.273	0.232	0.243	0.249	0.232	0.290	0.263	0.219	0.257	0.239	0.243	0.265	0.254	0.268	0.201	0.236	0.265		0.013	0.015	0.013
21	0.258	0.233	0.271	0.224	0.258	0.251	0.250	0.265	0.254	0.224	0.229	0.219	0.206	0.245	0.258	0.289	0.207	0.210	0.260	0.219		0.014	0.014
22	0.219	0.215	0.252	0.246	0.274	0.264	0.281	0.298	0.283	0.264	0.241	0.271	0.257	0.278	0.244	0.263	0.246	0.274	0.309	0.275	0.260		0.015
23	0.238	0.244	0.269	0.232	0.234	0.234	0.249	0.261	0.249	0.205	0.243	0.206	0.221	0.241	0.246	0.268	0.194	0.200	0.270	0.231	0.222	0.259	

Upper triangle: Standard deviation; Lower triangle: Kimura two-parameter distance. 1: *P. longicornis*; 2: NC012765.1 *Chrysochroa fulgidissima*; 3: NC013580.1 *Acmaeodera_*sp.; 4: NC060322.1 *Trachys variolaris*; 5: NC060321.1 *Coraebus cavifrons*; 6: NC046045.1 *Trachys auricollis*; 7: NC064327.1 *Meliboeus sinae*; 8: NC064326.1 *Coraebus diminutus*; 9: NC064325.1 *Coraebus cloueti*; 10: NC064324.1 *Agrilus sichuanus*; 11: NC057197.1 *Melanophila acuminata*; 12: NC081980.1 *Agrilus zanthoxylumi*; 13: NC080317.1 *Sambus kanssuensis*; 14: NC080316.1 *Cantonius szechuanensis*; 15: NC071967.1 *Julodis variolaris*; 16: NC071966.1 *Ptosima chinensis*; 17: NC071932.1 *Agrilus adelphinus*; 18: NC069980.1 *Agrilus discalis*; 19: NC067866.1 *Endelus continentalis*; 20: NC064400.1 *Agrilus ornatus*; 21: NC064328.1 *Sambus femoralis*; 22: NC062816.1 *Chalcophora japonica*; 23: NC030758.1 *Agrilus planipennis.*

## Data Availability

The original contributions presented in this study are included in the article/Supplementary Material. Further inquiries can be directed to the corresponding authors.

## Supplementary Materials

The following supporting information can be downloaded at: https://www.mdpi.com/article/10.3390/insects16080839/s1.

## Abbreviations

The following abbreviations are used in this manuscript:

FAF	Fujian Academy of Forestry, Fuzhou, China
NACRC-IOZ	National Animal Collection Resource Center, Institute of Zoology, Chinese Academy of Sciences, Beijing, China

## References

[1] Deyrolle H. (1864). Description des Buprestides de la Malaisie recueillés par M. Wallace pendant son voyage dans cet Archipel. Annales de la Société Entomologique de Belgique.

[2] Bílý S. (1993). A revision of the genera *Philanthaxia* Deyrolle and *Pagdeniella* Théry (Coleoptera, Buprestidae). Folia Heyrovskyana.

[3] Bílý S. (2001). Comments on the genus *Philanthaxia*, with descriptions of new species (Coleoptera: Buprestidae). Folia Heyrovskyana.

[4] Bílý S. (2006). *Philanthaxia nelsoni* sp. nov. from Indonesia (Coleoptera: Buprestidae). Pan-Pac. Entomol..

[5] Ong U., Hattori T. (2019). Jewel Beetles of Taiwan.

[6] Ong U., Curletti G., Hattori T. (2023). Jewel Beetles of Taiwan.

[7] Bílý S., Nakládal O. (2011). Four new species of the genus *Philanthaxia* Deyrolle, 1864 from Southeast Asia and comments on *P*. *iris* Obenberger, 1938 (Coleoptera, Buprestidae, Thomassetiini). ZooKeys.

[8] Bellamy C.L. (2008). A World Catalogue and Bibliography of the Jewel Beetles (Coleoptera: Buprestoidea).

[9] Bílý S. (2016). Four new species of the genus *Philanthaxia* Deyrolle, 1864 (Coleoptera: Buprestidae: Thomassetiini). Zootaxa.

[10] Ohmomo S. (2011). Notes on Buprestid Beetles (Coleoptera, Buprestidae) from Thailand, IX. A Key to the Species of the Genus *Philanthaxia* DEYROLLE, 1865 from Thailand, with Description of a New Species. Elytra Tokyo New Ser..

[11] Weidlich M. (1987). Systematik und Taxonomie der Buprestidae des mitteleozänen Geiseltales (Insecta, Coleoptera). Hallesches Jahrb. Geowissenschafen.

[12] Qi Z., Ai H., He X., Su R., Cai S., Song H. (2023). A study of *Anthaxia* subgen. *Thailandia* Bílý, 1990 from China (Coleoptera, Buprestidae, Buprestinae). ZooKeys.

[13] Folmer O., Black M., Hoeh W., Lutz R., Vrijenhoek R. (1994). DNA primers for amplification of mitochondrial cytochrome c oxidase subunit I from diverse metazoan invertebrates. Mol. Mar. Biol. Biotechnol..

[14] Evans A., Mckenna D., Bellamy C., Farrell B. (2015). Large-scale molecular phylogeny of metallic wood-boring beetles (Coleoptera: Buprestoidea) provides new insights into relationships and reveals multiple evolutionary origins of the larval leaf-mining habit. Syst. Entomol..

[15] Volkovitsh M. (2001). The comparative morphology of antennal structures in Buprestidae (Coleoptera): Evolutionary trends, taxonomic and phylogenetic implications. Part 1. Acta Musei Morav. Sci. Biol. [Bron].

[16] Huang X., Chen B., Wei Z., Shi A. (2022). First report of complete mitochondrial genome in the tribes *Coomaniellini* and *Dicercini* (Coleoptera: Buprestidae) and phylogenetic implications. Genes.

[17] Wei Z., Huang X., Shi A. (2023). First mitochondrial genome of subfamily *Julodinae* (Coleoptera, Buprestidae) with its phylogenetic implications. ZooKeys.

